# It Can’t Happen Here

**DOI:** 10.3201/eid1501.081327

**Published:** 2009-01

**Authors:** Marcela Natiello

**Affiliations:** Hospital “F. J. Muñiz,” Buenos Aires, Argentina

A banal virosis, no doubt
Fever fades away, back at work
I feel weary, I’ll be all right, I’m sure
What, now? My body itches
Could this be an allergy?
A pill to halt the itching
Again ill, what does this nausea mean?
Could this be dengue?
It’s on the borders, the global warming …
And yet, I didn’t leave the city
In Buenos Aires you can’t …
Just in case, I have a test done
I can’t believe it, the test is positive
Spread the news, could be more cases,
— Needs confirmation, it can’t happen here
Why not? Imported cases, *Aedes* thriving …
There must be others, we must warn people
— We are not sure, can’t happen here
At last, new test confirms. You must warn people
Not even now? What about the others?
Those who don’t know, they are at risk
— It won’t spread, can’t happen here
As a physician I feel responsible
Please spread the news
It did happen here!

